# Nutritional and Physicochemical Properties of Wild Lingonberry (*Vaccinium vitis-idaea* L.)—Effects of Geographic Origin

**DOI:** 10.3390/molecules28124589

**Published:** 2023-06-06

**Authors:** Dalia Urbonaviciene, Ramune Bobinaite, Pranas Viskelis, Jonas Viskelis, Aistis Petruskevicius, Viktorija Puzeryte, Laima Cesoniene, Remigijus Daubaras, Linards Klavins, Ceslovas Bobinas

**Affiliations:** 1 Institute of Horticulture, Lithuanian Research Centre for Agriculture and Forestry, 54333 Babtai, Lithuania; ramune.bobinaite@gmail.com (R.B.); jonas.viskelis@lammc.lt (J.V.); aistis.petruskevicius@lammc.lt (A.P.); viktorija.puzeryte@lammc.lt (V.P.); ceslovas.bobinas@lammc.lt (C.B.); 2 Botanical Garden, Vytautas Magnus University, 46324 Kaunas, Lithuania; laima.cesoniene@vdu.lt (L.C.); remigijus.daubaras@vdu.lt (R.D.); 3 Department of Environmental Science, University of Latvia, 1004 Riga, Latvia; linards.klavins@lu.lv

**Keywords:** wild population, bioactive compounds, physicochemical properties, different locations, authentication, liquid chromatography, *Vaccinium vitis-idaea* L.

## Abstract

In recent years, much attention has been devoted to *Vaccinium* L. berries because of their substantial potential to be adapted for the development of innovative food and pharmaceutical applications. The accumulation of plant secondary metabolites is extremely dependent on climate and other environmental conditions. In order to increase the reliability of the findings, this study was conducted with samples collected in four regions in Northern Europe (Norway, Finland, Latvia, and Lithuania) and analyzed in a single laboratory using a standardized methodology. The study aims to provide a comprehensive understanding of the nutritional (biologically active compounds (phenolic (477–775 mg/100 g fw), anthocyanins (20–57 mg/100 g fw), pro-anthocyanidins (condensed tannins (141–269 mg/100 g fw)) and antioxidant activity in different systems (ABTS^•+^, FRAP). Physicochemical properties (acidity, soluble solids, color) of wild *Vaccinium vitis-idaea* L. were also evaluated. The results may contribute to the development of functional foods and nutraceuticals with potential health benefits in the future. To the best of our knowledge, this is the first comprehensive report on the evaluation of the biologically active compounds of wild lingonberries from different Northern European countries based on one laboratory’s validated methods. The results indicated a geomorphological influence on the biochemical and physicochemical composition of wild *Vaccinium vitis-idaea* L. depending on their place of geographical origin.

## 1. Introduction

Landscape and climate have a significant impact on the properties of fruits. Many polyphenolic compounds are synthesized as a survival adaptation in response to external abiotic stresses [1]. Due to this adaptation to stresses such as cool climate, many of the fruits growing in the northern hemisphere are very beneficial to the human diet. Most of the berries from the *Vaccinium* genus are very rich in polyphenolics, such as anthocyanins. Wild lingonberry (*Vaccinium vitis-idaea* L.) is a small evergreen shrub that is common in the northern parts of Europe and Asia [2]. Lingonberry fruits are small red berries that have a unique taste. Due to their medicinal properties, historically, they have been used in traditional medicine to treat fever and oral and urinary tract infections [3]. Lingonberry fruit extracts have also been proven to be highly effective antimicrobial agents [4,5]. Lingonberries represent one of the most important and high-valued groups of modern-day “superfoods”. Dietary consumption of these berries has been recognized to be beneficial for human health for a long time. In addition to being delicious, these berries are rich in nutrients, minerals, vitamins (A, B1, B2, B3, C, and others), and several bioactive compounds, including phenolic antioxidants such as arbutin, catechin, anthocyanidins and procyanidins, phenolic acids, and others [6,7]. Accumulation of these compounds varies depending on the fruit development stage. Compounds such as anthocyanins reach the accumulation peak when the berry is fully ripe, while proanthocyanins, flavonols, and arbutin derivatives are found in the highest concentrations during the earlier stages of development [8].

The findings of various studies suggest that proanthocyanins, anthocyanins, and phenolic compounds possess a wide range of biological activities, including antioxidant, anti-inflammatory, antimicrobial, anticancer, cardioprotective, neuroprotective, and anti-diabetic effects [9,10,11]. Although further research is needed to fully understand the mechanisms of these compounds and optimize their therapeutic potential, the available evidence suggests that proanthocyanins are promising as natural compounds for the prevention and treatment of various diseases.

The world is likely to face a demographic crisis due to aging populations. The percentage of the global population that is 65 years of age and older is expected to rise from 10% to 16% by 2050 [12]. In developed countries, there is an increase in illnesses that are linked with old age, such as cardiovascular diseases and decreased cognitive abilities [13]. Due to the current global trends, it is likely that such illnesses will become even more prevalent in the future. Physical decline is partially caused by sedentary lifestyle and the regular consumption of food that lacks necessary nutrients [14]. As the portion of the global population that reaches old age is increasing and the benefits of healthy nutrition to both physical and mental condition are evident, the interest in practices that delay body deterioration also grows in importance. As such, foods containing antioxidants and anti-inflammatory compounds become increasingly relevant. Production of natural nutraceuticals made of fruits that are rich in such compounds may prove to be an effective and viable strategy due to the ever-growing nutraceutical market [15].

Lingonberries have been studied for their high antioxidant compounds, such as anthocyanins, phenolics, and flavonoids; however, most of the studies analyzing lingonberries’ phytochemical composition are conducted locally [16,17,18]. The authors of these studies acknowledge that plant secondary metabolite accumulation is substantially impacted by temperature, geographical latitude, and altitude, and opt to conduct the research in a small area in order to minimize the impact of environmental factors on the research results [16,18]. Since the accumulation of plant secondary metabolites is extremely dependent on environmental conditions, restricting the geographic scope of the study may also limit the applicability of the findings from studies conducted in different regions due to the divergence in the experimental methods and laboratory protocols employed across different countries. This study was conducted using one method in a single research facility, utilizing samples collected in four regions in Northern Europe (Norway, Finland, Latvia, and Lithuania), and aims to provide a comprehensive understanding of the nutritional and physicochemical properties of wild lingonberry, which in the future may contribute to the development of functional foods and nutraceuticals with potential health benefits.

## 2. Results and Discussion

The variation of physicochemical properties was analyzed in naturally grown (wild) lingonberries gathered from different sites in Northern Europe (Latvia (LVA), Lithuania (LTU), Norway (NOR), and Finland (FIN)). During the study, traditional quality parameters were analyzed, including soluble solids (SS), acidity levels, and biologically active compounds such as pigments. The quality of lingonberries is closely linked to their soluble solids content and acidity. Total acids, indicated by the measured pH level, contribute to the berries’ sweetness and acidity, whereas the evaluation of soluble solids reveals the sugar levels and titratable acids of both the berries and their products [19,20].

**Figure 1:**
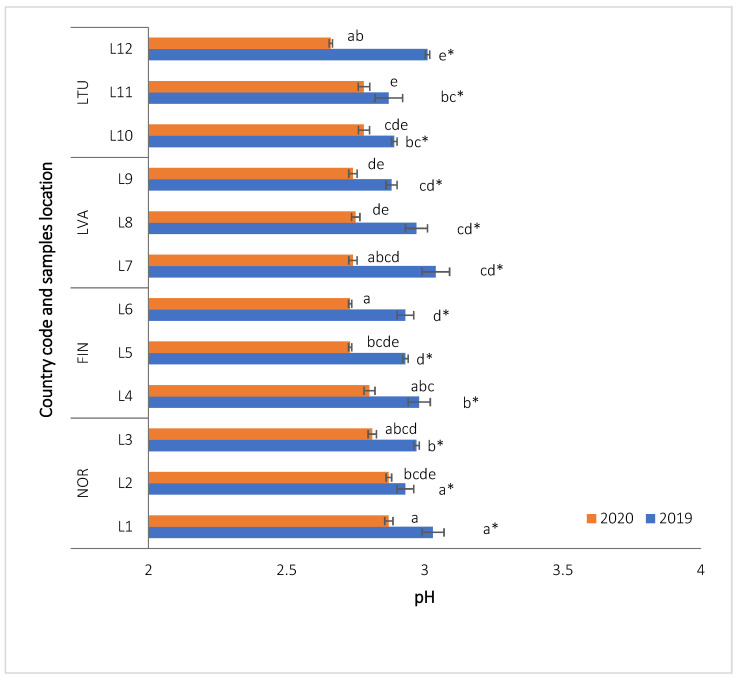
pH values of wild *Vaccinium vitis-idaea* L. from different countries and locations. Note: Values are presented as means ± standard deviation. Different letters within the same column indicate significant differences between the collection locations (L1–12) (*p* < 0.05). Significant differences between 2019 and 2020 are indicated by asterisks (*) (*p* < 0.05).

The results of the pH and total soluble solids (°Brix) analysis in lingonberries (Figure 1) showed that the pH levels ranged from 2.66 to 3.03. In 2019, the mean pH levels of berries collected from different countries did not show any significant differences. The pH levels of lingonberries in this study were consistent with previous reports (2.74–2.90) [21]. In summary, our study did not find significant differences in the average pH values of the lingonberry populations in different countries (Figure 1). The change was within the margin of error and ranged about 4% (over 2 years, 2.74–2.98). The growth of berries and their sweetness may be affected by environmental factors such as temperature, day length, and light intensity, as suggested by studies conducted by Primetta et al. [22].

**Figure 2:**
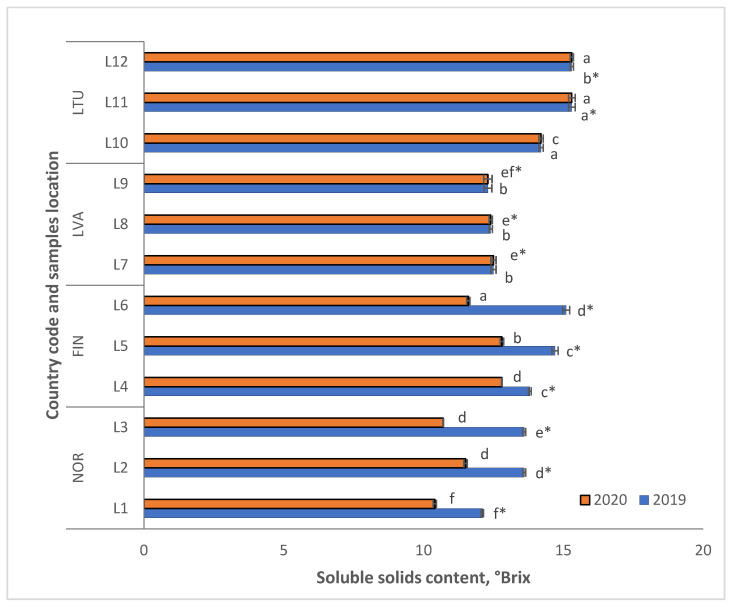
Soluble solids content of wild *Vaccinium vitis-idaea* L. from different countries and locations. Note: Values are presented as means ± standard deviation. Different letters within the same column indicate significant differences between the collection locations (L1–12) (*p* < 0.05). Significant differences between 2019 and 2020 are indicated by asterisks (*) (*p* < 0.05).

The data demonstrate that lingonberries grown in Lithuania and Finland had higher levels of soluble solids (SS) in 2020 compared to the previous year (2019). However, the increase was only 1.1 and 1.2 times higher, respectively, as shown in Figure 2. In 2020, Norwegian lingonberries had the lowest average SS content. In contrast, the highest average SS content was found in lingonberries from Lithuania locations harvested over two years (2019–2020), with average values of 14.9 and 13.7, respectively. Meanwhile, the lowest average SS value was found for the lingonberries from Norway (13.1 and 10.9, in 2019 and 2020, respectively) (Figure 2). The SS content of the lingonberry measurements ranged from 10.4 to 15.3 °Brix, as shown in Figure 2. In 2019, the lingonberries grown in LTU and FIN had higher SS levels, while in 2020, the berries from LTU and LVA had higher SS contents (Figure 2). The wild lingonberries from NOR, FIN, and LTU (L11 and L12) had significantly lower SS content in 2020 when compared to the findings from 2019.

**Figure 3:**
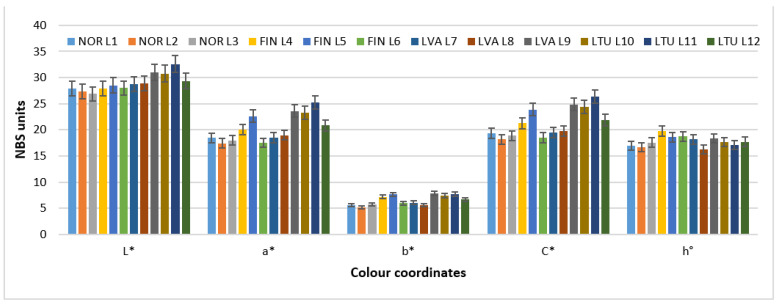
Average of color coordinates of 2019 and 2020 in wild lingonberries. Note: Values are presented as means ± standard deviation. * the color coordinates are expressed in NBS units.

Color is a very important sensorial parameter in fruit analysis. It can serve as a key indicator for certain valuable secondary metabolites, such as anthocyanins. A difference between wild lingonberry color coordinates was not detected in the results from two years of analysis in different locations (Figure 3). Figure 3 presents the average values obtained over a period of two years, in different locations and countries.

**Figure 4:**
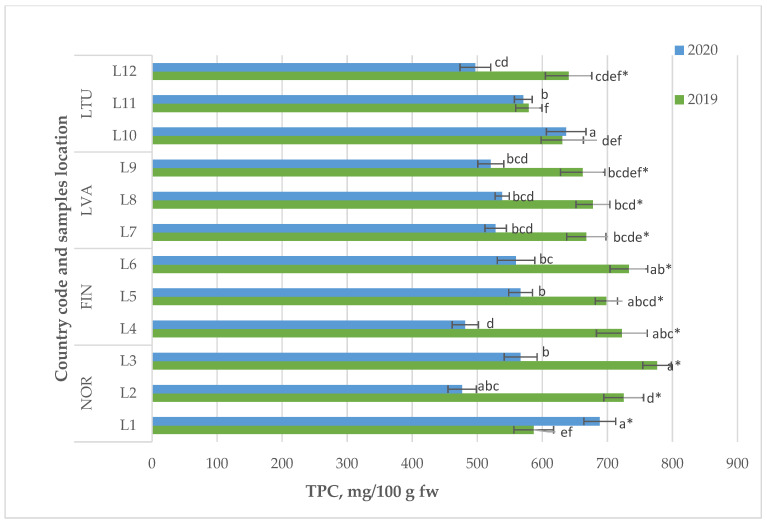
Total phenolics content of *Vaccinium vitis-idaea* L. Note: Values are presented as means ± standard deviation. Different letters within the same column indicate significant differences between the collection locations (L1–12) (*p* < 0.05). Significant differences between 2019 and 2020 are indicated by asterisks (*) (*p* < 0.05).

The study of total phenolic compounds as the main biologically active compounds of *Vaccinium vitis-idaea* L. revealed that lingonberry samples from NOR and FIN exhibited the highest TAC in both 2019 and 2020, ranging from 41.7 to 57.0 mg/100 g in fresh weight (fw). Berry samples from LVA followed suit, with a TAC of 29.4 to 32.9 mg/100 g fw, while LTU had the lowest TAC values, ranging from 19.8 to 27.4 mg/100 g fw (Figure 4). Additionally, the study noted significant annual variations in the TAC of lingonberry samples obtained from some locations, including L1, L2, L3, and L5.

Based on various studies, fruits can be categorized by evaluating their total phenolic content (TPC), with TPC values falling into three categories: low (<100 mg/100 g), medium (100–500 mg/100 g), and high (>500 mg/100 g). Berries have been found to be an excellent source of these beneficial compounds, according to research conducted by Vasco et al. [23]. The total phenolic content of the examined lingonberry samples matched previously documented results. For instance, the TPC of lingonberries cultivated in the central region of Poland varied from 468 to 661 mg/100 g fw, according to Dróżdż et al. [16]. Similarly, lingonberry varieties grown in Oregon (United States) had comparable values, ranging from 431 to 660 mg/100 g fw, as measured by Lee and Finn [21]. Meanwhile, Dincheva and Badjakov [24] reported higher TPC levels (ranging from 714 to 791 mg/100 g fw) in lingonberries found in natural habitats in Bulgaria. 

During 2019, the lingonberry samples with the highest TPC values were obtained from the Northern European countries NOR and FIN. However, in 2020, the average TPC values were found to be similar in samples collected from all four countries included in the study (Figure 4). Additionally, in 2020, the average TPC values for lingonberries from NOR, FIN, and LVA were significantly lower compared to those determined in 2019. A decrease of the average TPC values in lingonberry by up to 25% was observed in the results obtained in 2020, indicating a significant impact of suboptimal weather conditions on the accumulation of phenolic compounds.

The total phenolic content (TPC) of the *Vaccinium vitis-idaea* L. samples varied from 477 (NOR L2 in 2020) to 776 mg/100 g fw (NOR L3 in 2019) according to Figure 4.

**Figure 5:**
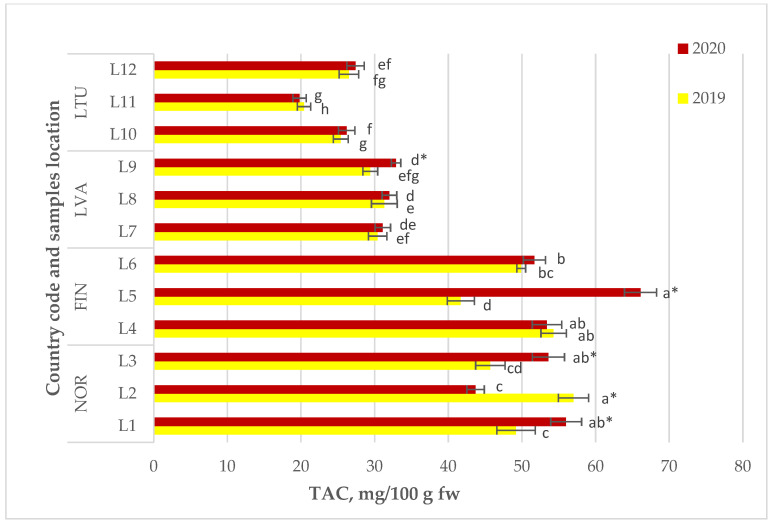
Total anthocyanins content of wild *Vaccinium vitis-idaea* L. Note: Values are presented as means ± standard deviation. Different letters within the same column indicate significant differences between the collection locations (L1–12) (*p* < 0.05). Significant differences between 2019 and 2020 are indicated by asterisks (*) (*p* < 0.05).

The lingonberry samples showed a range of 19.8 to 57.0 mg/100 g fw for their total anthocyanin content, as indicated in Figure 5. Although flavan-3-ols (catechin and epicatechin) and flavonols (mainly quercetin glycosides) are the primary flavonoids in lingonberries [25,26], it is noteworthy that anthocyanins are not the main flavonoids in lingonberries. However, anthocyanins are very important in the human diet. As discussed earlier, these compounds may have antimicrobial, antiviral (against the SARS-CoV-2 virus) [27] and antioxidative [28] properties. The TAC levels were analyzed in samples gathered from NOR, FIN, LVA, and LTU in both 2019 and 2020. Among these, the NOR and FIN samples had the highest TAC levels, ranging from 41.7 to 57.0 mg/100 g fw. LVA and LTU followed, with TAC levels ranging from 29.4 to 32.9 mg/100 g fw and 19.8 to 27.4 mg/100 g fw, respectively (as shown in Figure 5). The study also noted significant annual variation in TAC levels of lingonberry samples collected from specific locations (L1, L2, L3, and L5). 

**Figure 6:**
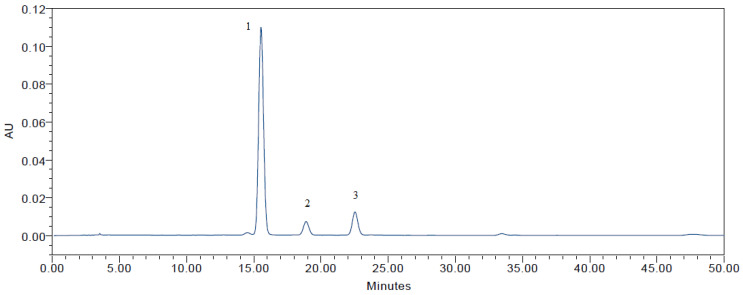
The anthocyanin profile determined by HPLC-DAD in wild *Vaccinium vitis-idaea* L. Peaks: (1) cyanidin-3-O-galactoside (C-3-Gal), (2) cyanidin-3-O-glucoside (C-3-Glu), and (3) cyanidin-3-O-arabinoside (C-3-Ara).

Wild *Vaccinium vitis-idaea* L. samples were analyzed using HPLC-DAD, and three main anthocyanins (cyanidin-3-O-galactoside (C-3-Gal), cyanidin-3-O-glucoside (C-3-Glu), and cyanidin-3-O-arabinoside (C-3-Ara)) were identified, as shown in Figure 6. Among the anthocyanins we identified, cyanidin-3-O-galactoside was the most prevalent, constituting 83.5% of the total anthocyanin content. The lingonberry anthocyanins profile was compared with individual standards (C-3-Gal, C-3-Glu, and C-3-Ara), and with the European Pharmacopoeia Reference Standards and the United States Pharmacopeia, of the main anthocyanins found in berries (the reference standard is Bilberry dry extract CRS) [29,30] (Figures S1–S5 in the Supplementary Materials). Our findings are consistent with prior research by Kähkönen et al. [31], Ek et al. [32], Lehtonen et al. [33], and Lätti et al. [34], which found that cyanidin-based anthocyanins are the only ones present in lingonberry samples. The dominant anthocyanin was cyanidin-3-O-galactoside (C-3-Gal), comprising 74.4% to 83.5% of the total anthocyanins present, which is also consistent with the results of other studies [21,31,32,34,35].

**Figure 7:**
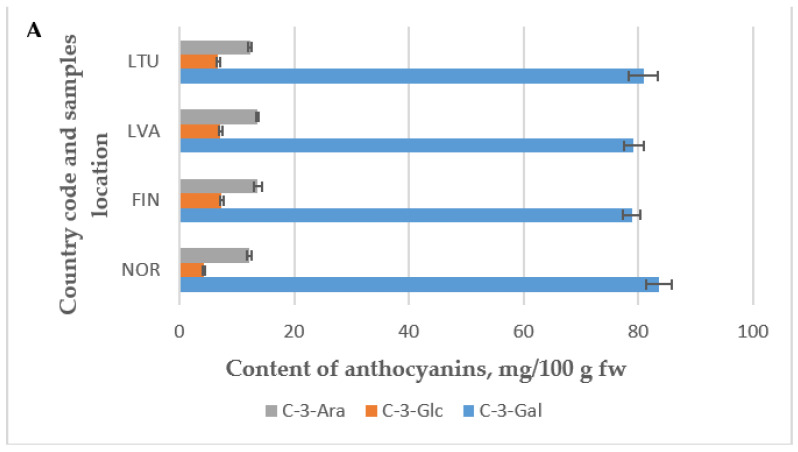

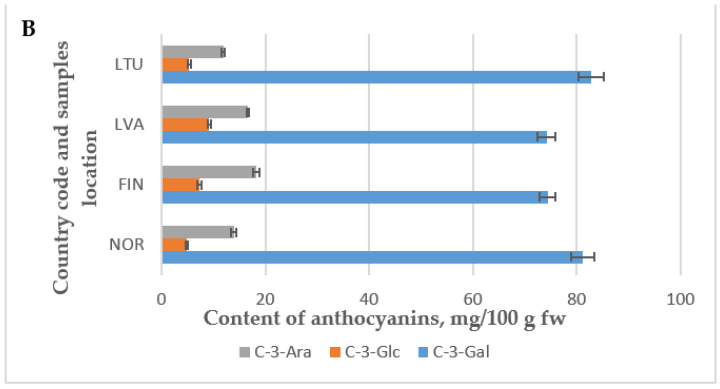
Individual anthocyanins content in wild *Vaccinium vitis-idaea* L. in 2019 (**A**) and 2020 (**B**).

Concentrations of other anthocyanins in the investigated lingonberries consisted of 12.2% to 18.6% cyanidin-3-O-arabinoside (C-3-Ara) and 4.3% to 9.7% cyanidin-3-glucoside (C-3-Glu). Figure 7A,B show the average percentage values of each anthocyanin in lingonberries harvested in different counties during 2019 and 2020. Foley and Debnath [28] previously reported a similar distribution of anthocyanins in lingonberries, which consisted of 84% cyanidin-3-O-galactoside (C-3-Gal), 5% cyanidin-3-O-glucoside (C-3-Glu), and 11% cyanidin-3-O-arabinoside (C-3-Ara). The anthocyanin profile of the investigated lingonberries was comparable to previous reports by Kähkönen et al. [31], Lee and Finn [21], and Isaak et al. [36], but without the additional minor peaks noted by Ek et al. [32] and Lätti et al. [34]. The samples of lingonberry collected from Norway, Finland, Latvia, and Lithuania showed comparable levels of the identified anthocyanins, specifically cyanidin-3-O-galactoside (C-3-Gal), cyanidin-3-O-glucoside (C-3-Glu), and cyanidin-3-O-arabinosidev (C-3-Ara), in 2019 (Figure 7A) and 2020 (Figure 7B).

Pro-anthocyanidins are part of the plants’ biologically active compounds [37,38]. The results of the pro-anthocyanidin content investigation from three different locations in Lithuania, Latvia, Norwegian, and Finland in 2019 and 2020 were analyzed (Table 1). When evaluating the data, it was noticeable that the average pro-anthocyanidins content ranged from 141.3 to 268.6 mg/100 g fw. The study also noted significant annual variation in the lingonberry samples collected from specific locations (L3, L4, L10, and L12).

The levels of vitamin C in berries collected from different countries did not show any significant differences between the years 2019 and 2020. Vitamin C contents ranged from 8.8 to 9.6 mg/100 g fw (Table 2).

**Figure 8:**
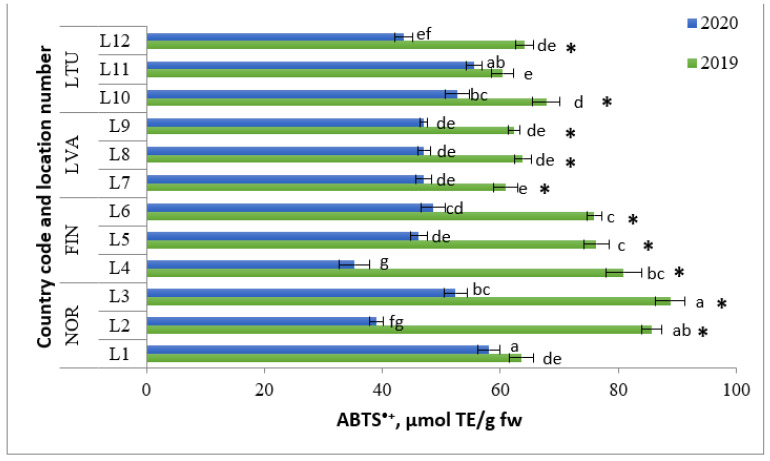
ABTS antioxidant assay of lingonberries (μmol TE/g fw). Note: Different letters above the same color bars indicate significant differences between the mean values (*p* < 0.05). Significant differences between 2019 and 2020 are indicated by asterisks (*) (*p* < 0.05).

Antioxidant activity in wild *Vaccinium vitis-idaea* L. berries was analyzed using ferric-reducing antioxidant activity (FRAP) and the 2,2-azinobis (3-ethylbenzothiazoline-6-sulfonic acid) assay (ABTS^•+^). The radical scavenging activity (RSA) of extracts was evaluated using the ABTS^•+^ radical cation assay. According to Grace et al. [39], species derived from *Vaccinium*, especially lingonberry, possess remarkable antimicrobial and antioxidant properties. Lingonberries were found to have an ABTS^•+^ RSA ranging from 35.3 μmol TE/g fw (FIN L4 in 2020) to 88.8 μmol TE/g fw (NOR L3 in 2019) (Figure 8). In 2019, the berries from NOR had the highest ABTS^•+^ RSA values, followed by those from FIN, whereas in 2020, NOR and LTU berries exhibited the highest values. 

**Figure 9:**
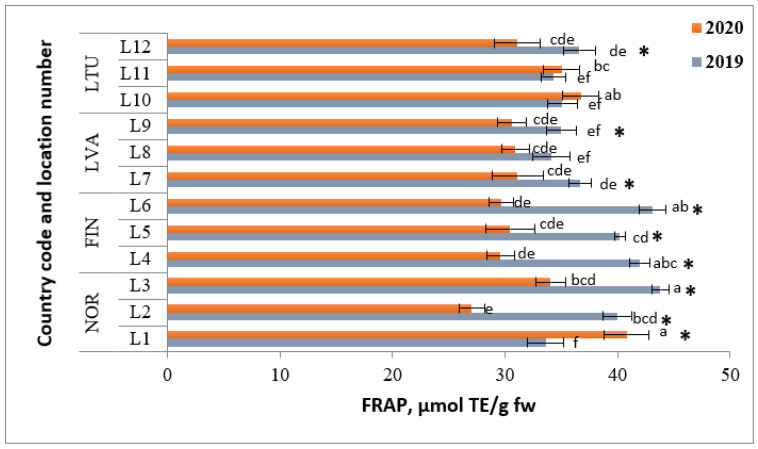
FRAP antioxidant assay of lingonberries (μmol TE/g fw). Note: Different letters above the same color bars indicate significant differences between the mean values (*p* < 0.05). Significant differences between 2019 and 2020 are indicated by asterisks (*) (*p* < 0.05).

The FRAP values of lingonberries varied from 32.6 (NOR L1) to 43.8 μmol TE/g fw (NOR L3) in 2019, whereas the values obtained during the analysis of samples collected in 2020 were lower and ranged from 27.1 (NOR L2) to 40.8 μmol TE/g fw (NOR L1) (as shown in Figure 9). The berry samples collected in FIN had the highest mean FRAP value in 2019 (41.4 μmol TE/g fw), followed by the samples from NOR (38.8 μmol TE/g fw). In 2020, berries gathered in LTU and NOR had the highest FRAP values (34.3 and 34.0 μmol TE/g fw, respectively).

Correlations were derived when comparing the data of this study. The investigation of lingonberries found a strong positive correlation (R = 0.93 and 0.92 for FRAP and ABTS^•+^ assays, respectively) between total phenolic content (TPC) and antioxidant activity. Interestingly, no correlation was observed between total anthocyanin content (TAC) and antioxidant activity, suggesting that other phenolic compounds significantly contribute to the berries’ antioxidant properties, while anthocyanin’s impact on these properties may be minimal. These findings align with a previous study by Nestby et al. [40], who reported no correlation between antioxidant activity and anthocyanin content in 18 lingonberry genotypes. However, the study also highlights the potential cardioprotective effects of the three anthocyanins in lingonberries, namely cyanidin-3-O-galactoside, cyanidin-3-O-glucoside, and cyanidin-3-O-arabinoside, which have been shown to protect cardiac cells from oxidative stress-induced apoptosis when used as a dietary supplement [36].

**Figure 10:**
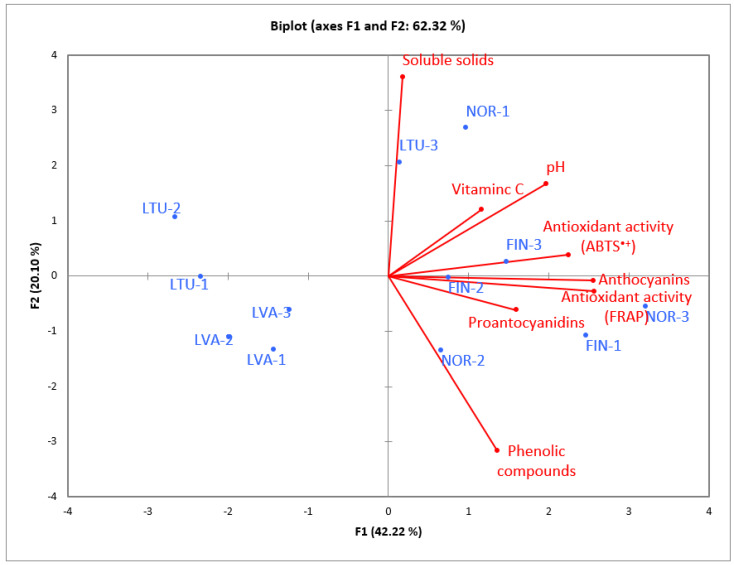
Principal component analysis (PCA) biplot for the relationships between different sample locations and the nutritional and physicochemical properties of wild lingonberry (*Vaccinium vitis-idaea* L.).

The principal component analysis shows which different locations can be characterized by which chemical composition parameter (Figure 10). Lithuanian and Latvian *Vaccinium vitis-idaea* L. can be grouped separately from other locations, while lingonberries from Norway and Finland can be better characterized by their nutritional and physicochemical properties.

## 3. Materials and Methods

### 3.1. Sample Preparation

The ripe wild population berries of *Vaccinium vitis-idaea* L. were handpicked during the summer of 2019 and 2020 at the time periods when they are typically harvested for commercial purposes. Sampling locations for the populations investigated together with the geographical coordinates are shown in Table 3. Wild populations of lingonberries were sampled in three locations across Baltic and Nordic regions in typical growing areas, including Finland, Norway, Latvia, and Lithuania. The plants were visually examined to determine if they were fully pigmented and undamaged. The plants were identified according to the morphological characteristics by Laima Cesoniene and Remigijus Daubaras.

The *Vaccinium vitis-idaea* L. samples were quickly cooled below +10 °C before being frozen and kept at −55 °C until the analysis was conducted within the next month.

### 3.2. Sample Preparation

The lingonberry sample was homogenized for 5 min using a Polytron (PT 1200E) from Kinematica, located in Luzern, Switzerland. Post-homogenization, 5000 g of the sample was extracted using an acidified (0.5% HCl) aqueous ethanol solution (70% *v*/*v*) of 40 mL. The sample was then filtrated using a Buchner funnel and a Whatman No. 1 filter paper to ensure the absence of impurities. The filtered sample was stored at a temperature of 4 ± 1 °C until the analysis. The extraction process was carried out at 23 °C for a period of 24 h, with steady shaking at 150 rpm. 

### 3.3. Determination of Physicochemical Parameters

#### 3.3.1. Measurement of pH and °Brix

The pH was determined using the pH meter model MW102 with MA 920 electrodes (Milwaukee, Sat Baciu, Romania). The soluble solids (SS) content, in °Brix, was obtained with an ‘Abbe’ digital refractometer, model DR-A1 (ATAGO Co., Bellevue, WA, USA), at 22 °C.

#### 3.3.2. Determination of Ascorbic Acid

The determination of ascorbic acid, commonly known as vitamin C, was carried out utilizing a titrimetric approach in accordance with AOAC guidelines [41]. To account for the presence of strong pigmentation in the extracts, chloroform was used. A solution of 2,6-dichlorophenolindophenol sodium salt was used as the titrant. 

#### 3.3.3. Evaluation of Color in Lingonberries

Lingonberries’ color indices were measured with the spectrophotometer MiniScan XE Plus (Hunter Associates Laboratory, Reston, USA). Regarding light reflection, the parameters L*, a*, and b* (lightness and indices of redness and yellowness, respectively, according to the CIE L*a*b* scale) were measured, and chroma (C = (a*^2^ +b*^2^)^1/2^) and the hue angle (h° = arctan(b*/a*)) were calculated. The spectrophotometer was calibrated before each set of measurements using a light catcher and a white color standard. The color coordinates XYZ were: X = 81.3, Y = 86.2, and Z = 92.7, in color space. The color coordinates of the CIE L*a*b* scale were recorded as L*, which represents the ratio of white to black, a*, which is the ratio of red (a* > 0) to green (a* < 0), and b*, which represents the ratio of yellow (b* > 0) to blue (b* < 0). The software “Universal Software V.4-10” (Hunter Associates Laboratory, Reston, VA, USA) was used to process the color parameters. At least three different measurements were carried out for every sample.

### 3.4. Analysis

#### 3.4.1. Determination of Total Phenolics Content (TPC)

The total phenolics content (TPC) of the lingonberry extracts was obtained using the Folin–Ciocalteu method, as previously reported by Bobinaite et al. [42]. In short, the test tubes were filled with 1.0 mL of appropriately diluted extract and mixed with 5.0 mL of Folin–Ciocalteu’s phenol reagent diluted in distilled water (1/10, *v*/*v*) and 4.0 mL of Na_2_CO_3_ (7.5%). The test solution was left to incubate for 60 min, protected from light exposure, and measured at 765 nm with a Genesys-10 UV/Vis spectrophotometer (Thermo Spectronic, Rochester, NY, USA). The results were presented in mg of gallic acid equivalents (GAE) per 100 g of berries fresh weight (FW). To calculate the results, we used gallic acid as the standard for the calibration curve.

#### 3.4.2. HPLC Analysis of Total Anthocyanins Content (TAC)

The Waters 2695 series HPLC system, outfitted with the Waters 2998 photodiode array detector (DAD) (Waters Corporation, Milford, MA, USA), was used to evaluate anthocyanins, as previously reported by Urbonaviciene et al. [43]. Analytical separation was performed using a LiChroCART Purospher^®^ STAR RP18 end-capped column (250 × 4.6 mm, 5 µm particle size), with the guard column Purospher STAR RP18e (4.0 × 4.0 mm, 5 µm (Merck KgaA, Darmstadt, Germany)). The column oven was set to a temperature of 25 °C. Eluents A and B comprised the mobile phase and were aqueous 10% formic acid and ACN-MeOH (85:15, *v*/*v*), respectively. The gradient program was as follows: 0–2 min 4–6% eluent B; 2–4 min 6–8% eluent B; 4–12 min 8–9% eluent B; 12–46 min 9–11% eluent B; 46–48 min 11–24% eluent B; 48–52 min 24–34% eluent B; 52–59 min 34–80% eluent B; 59–61 min 80–20% eluent B; 61–65 min 4% eluent B. The injection had a 10 µL volume.

To identify anthocyanins, the wavelength was set to 520 nm, and DAD data were collected between 200 and 600 nm. By comparing HPLC retention durations (RT) and the UV absorbance maximum with commercial standards or published data, anthocyanins present in lingonberry extracts were identified. The external standards of cyanidin-3-O-galactoside (C-3-Gal), cyanidin-3-O-glucoside (C-3-Glu), and cyanidin-3-O-arabinoside (C-3-Ara), and the anthocyanins chromatography reference standard (CRS) of Bilberry dry extract of Pharmacopoeia, purchased from Sigma-Aldrich (Taufkirchen, Germany), were used. The establishment of a seven-point external standard calibration curve for cyanidin-3-O-galactoside, the commercial standard, was achieved through dilution in a solvent mixture composed of 10% solvent B and 90% solvent A. The concentration range of this curve was from 1 to 100 mg/L and its linearity was deemed acceptable, with an R^2^ value of 0.999. The total content of anthocyanins present in the berry extracts was calculated by determining the sum of individual quantities of the compounds as equivalents of cyanidin-3-O-galactoside (C-3-Gal) per 100 g of fresh weight (fw) of the berries.

#### 3.4.3. Determination of Total Pro-Anthocyanidins Content (TPC)

The DMAC (4-(dimethylamino)cinnamaldehyde) method was used to establish the total pro-anthocyanidins content [38]. For each sample, 3 mL of DMAC reagent solution was utilized, and 20 μL of the diluted extracts from wild lingonberries was added. The absorption was measured after 5 min with a spectrophotometer (model UV-1800, Shimadzu) at 640 nm against the reagent blank. Epicatechin (0.002–0.016 µg/mL) was used as the standard for the calibration curve. The results were expressed as epicatechin equivalents per 100 g of fresh weight of lingonberries (mg/100 g fw).

#### 3.4.4. Establishment of Ferric-Reducing Antioxidant Activity (FRAP)

The FRAP assay was carried out using the Benzie and Strain method [44], with a few minor adjustments [45]. To conduct the FRAP assay, a 0.3 M sodium acetate buffer (pH 3.6) was prepared by dissolving 3.1 g of sodium acetate and 16 mL of acetic acid in 1000 mL of distilled water. Additionally, 10 mM of 2,4,6-tripyridyl-s-triazine (TPTZ) solution was prepared by dissolving 0.031 g of TPTZ in 10 mL of 40 mM of HCl, and 20 mM of ferric solution was prepared by dissolving 0.054 g of FeCl_3_·6H_2_O in 10 mL of distilled water. These solutions were thoroughly mixed to create the working FRAP reagent in the proportion of 10:1:1. For the analysis, 20 µL of diluted extract was combined with 2 mL of freshly made FRAP working solution, and the mixture was incubated for 30 min at room temperature. The change in absorbance brought on by the reduction of the ferric-tripyridyltriazine (Fe III-TPTZ) complex by the antioxidants present in the samples was monitored at 593 nm using a Genesys-10 UV/Vis spectrophotometer (Thermo Spectronic, Rochester, NY, USA). Prior to and after each analysis, the absorbance of blank samples was measured using the same conditions. The antioxidant activity was determined from the change in absorbance. The antioxidant activity was measured using Trolox as the standard, and it was expressed as µmol of Trolox equivalents (µmol TE) per g of berries (fw). 

#### 3.4.5. Establishment of ABTS^•+^ Radical Scavenging Activity (ABTS RSA)

The radical scavenging activity (RSA) of extracts was evaluated using the ABTS^•+^ radical cation assay [46]. ABTS solution (2 mM) was prepared by dissolving 2,2’-azinobis(3-ethylbenzothiazoline-6-sulphonic acid) diammonium salt in 50 mL of phosphate-buffered saline (PBS), obtained by dissolving 8.18 g of NaCl, 0.27 g of KH_2_PO_4_, 1.42 g of Na_2_HPO_4_, and 0.15 g of KCl in 1 L of pure water. The pH of the prepared solution was adjusted to 7.4 using NaOH. Then, a 70 mM solution of K_2_S_2_O_8_ was made in pure water. A working solution (ABTS^•+^ radical cation) was established by combining 50 mL of ABTS solution with 200 μL of K_2_S_2_O_8_ solution and allowing the combination to stand for 15–16 h at room temperature in the dark before use. To determine the antiradical properties of the extracts, a 1 cm path length cuvette was used to mix 2 mL of ABTS^•+^ solution with 20 μL of the extract. After leaving the mixture in the dark at room temperature for 30 min, the absorbance of the reaction was measured at 734 nm. The antioxidant activity was measured using Trolox as the standard, and it was expressed as µmol of Trolox equivalents (µmol TE) per g of berries (fw).

### 3.5. Statistical Analysis

The experiments were carried out in triplicate. The data were presented as the mean value and the standard deviation. The software SPSS 20 (SPSS Inc., Chicago, IL, USA) was used to calculate the mean values and standard deviations of the obtained data. Significant differences between means were determined by one-way analysis of variance (ANOVA) and the post hoc Tukey’s HSD test. Differences were considered significant at *p* < 0.05. The relationships between different sample locations and the biochemical quality parameters were evaluated with the XLSTAT 2018 (New York, NY, USA) using principal component analysis (PCA).

## 4. Conclusions

A comprehensive report on the biologically active compounds, antioxidant activity, and physicochemical properties of the evaluation of wild *Vaccinium vitis-idaea* L. from different Northern European countries and different locations was presented. The natural variation in biologically active compounds needs to be investigated in order to establish fingerprints in the wild berries from northern countries in comparison to more southern natural habitats. The methods were considered suitable for fingerprint analysis to check the origin and control the quality of wild berries. The methods used here could also be used for the quality control of raw materials and during the process of production (in the pharmaceutical and food industries), such as for determining the quality and the origin, and especially in the detection of adulterants, which in the future may contribute to the development of functional foods and nutraceuticals with potential health benefits. The results of the evaluation of the biologically active compounds and physicochemical properties (acidity, soluble solids, color) indicated a significant geomorphological influence on the biochemical and physicochemical composition of wild *Vaccinium vitis-idaea* L. berries.

## Figures and Tables

**Table 1:** Total pro-anthocyanidins content in wild *Vaccinium vitis-idaea* L.

Country Code	Location Name	Pro-Anthocyanidins, mg/100 g fw	
		2019	2020
NOR	L1	141.3 ± 3.96 def	149.0 ± 3.57 a
	L2	180.6 ± 5.41 f	196.8 ± 4.07 b
	L3	208.0 ± 6.45 a *	191.8 ± 4.64 bc
	Mean	161.0 ± 6.71 a	179.2 ± 5.55 a *
FIN	L4	213.1 ± 3.02 bc	268.6 ± 2.58 g *
	L5	189.2 ± 2.18 c *	161.4 ± 1.48 de
	L6	182.8 ± 1.27 c	215.4 ± 2.08 cd *
	Mean	186.0 ± 3.12 a	215.1 ± 6.41 a *
LVA	L7	152.5 ± 2.01 e	171.8 ± 1.40 de *
	L8	153.1 ± 1.45 de	168.9 ± 1.21 de
	L9	155.2 ± 1.10 de	170.1 ± 0.58 de *
	Mean	153.6 ± 1.82 b	170.3 ± 0.91 a *
LTU	L10	196.2 ± 2.34 d	235.9 ± 0.58 de *
	L11	159.5 ± 1.52 de	212.4 ± 1.00 de *
	L12	200.7 ± 1.93 e	200.1 ± 1.47 ef
	Mean	159.5 ± 3.65 b	216.1 ± 5.56 a *

Note: Values are presented as means ± standard deviation. Different letters within the same column indicate significant differences between the collection locations (L1–12) (*p* < 0.05). Significant differences between 2019 and 2020 are indicated by asterisks (*) (*p* < 0.05).

**Table 2:** Content of vitamin C in wild *Vaccinium vitis-idaea* L.

Country Code	Location Name	Vitamin C, mg/100 g fw	
		2019	2020
NOR	L1	9.6 ± 0.28	9.2 ± 0.25
	L2	9.5 ± 0.41	8.8 ± 0.37
	L3	9.5 ± 0.36	9.1 ± 0.19
	Mean	9.5 ± 0.09	9.0 ± 0.21
FIN	L4	9.2 ± 0.22	8.9 ± 2.58
	L5	9.6 ± 0.18	9.2 ± 1.48
	L6	9.4 ± 0.27	9.1 ± 2.08
	Mean	9.4 ± 0.20	9.1 ± 0.15
LVA	L7	9.2 ± 0.20	8.8 ± 0.25
	L8	9.1 ± 0.35	8.9 ± 0.21
	L9	9.0 ± 0.11	9.1 ± 0,28
	Mean	9.1 ± 0.10	8.8 ± 0.07
LTU	L10	9.2 ± 0.34	8.8 ± 0.18
	L11	9.2 ± 0.32	9.0 ± 0.31
	L12	9.1 ± 0.18	8.9 ± 0.47
	Mean	8.9 ± 0.06	8.9 ± 0.10

**Table 3:** Collecting locations and countries of wild lingonberries.

Location Number	Country Code	Sample Coordinates	Province, Geographical Region
L1	NOR (Norway)	Lat. 69° 41.665200′; Lon. 18° 41.244726′	Hålogaland, Tromso distr.
L2	NOR (Norway)	Lat. 69° 45.144144′; Lon. 19° 1.097034′	Kvaloya, Tromso distr.
L3	NOR (Norway)	Lat. 69° 43.550706′; Lon. 19° 7.144680′	Knutsenkohen, distr.
L4	FIN (Finland)	Lat. 65° 02.4578′; Lon. 25° 42.8869′	Saviharju, Oulu reg.
L5	FIN (Finland)	Lat. 65° 00.6398′; Lon. 26° 05.5423′	Karahka, Oulu reg.
L6	FIN (Finland)	Lat. 64° 54.3698′; Lon. 25° 44.6767′	Hangaskangas, Oulu reg.
L7	LVA (Latvia)	Lat. 56° 26.397300′; Lon. 22° 48.768360′	Auce, Dobele distr.
L8	LVA (Latvia)	Lat. 56° 26.426580′; Lon. 22° 49.528800′	Auce, Dobele distr.
L9	LVA (Latvia)	Lat. 56° 26.659200′; Lon. 22° 49.423800′	Auce, Dobele distr.
L10	LTU (Lithuania)	Lat: 54° 5.188333′; Lon. 24° 39.823333′	Rudnia, Alytus distr.
L11	LTU (Lithuania)	Lat: 54° 45.385000′; Lon. 23° 25.430000′	Kazlu Ruda, Marijampole distr.
L12	LTU (Lithuania)	Lat: 55° 5.091667′; Lon. 22° 27.851667′	Viesvile, Jurbarkas distr.

## Data Availability

All data generated during this study are included in this article.

## Supplementary Materials

The following supporting information can be downloaded at: https://www.mdpi.com/article/10.3390/molecules28124589/s1, Figure S1: The anthocyanin profile determined by HPLC-DAD in wild Vaccinium vitis-idaea L. (A – NOR; B – LVA) Peaks: 1. Cyanidin-3-O-galactoside (C-3-Gal); 2. Cyanidin-3-O-glucoside (C-3-Glu); 3. Cya-nidin-3-O-arabinoside (C-3-Ara); Figure S2: HPLC-DAD profiles of anthocyanins of European and US Pharmacopoeia Cromatography Reference Standard (CRS) Bilberry dry extract at 520 nm; Figure S3: Integrated chromatogram; Figure S4: The external standards of cyaniding-3-O-galactoside (C-3-Gal), cyanidin-3-O-arabinoside (C-3-Ara) and sample of anthocyanin profile determined by HPLC-DAD in wild Vaccinium vitis-idaea L.; Figure S5. The external standard of cyanidin-3-O-glucoside (C-3-Glu) and sample of anthocyanin profile determined by HPLC-DAD in wild *Vaccinium vitis-idaea* L.
