# Supplementary material for: Nutritional and Physicochemical Properties of Wild Lingonberry (Vaccinium vitis-idaea L.)—Effects of Geographic Origin
Source: Molecules. 2023 Jun 6;28(12):4589. doi: 10.3390/molecules28124589 (PMC10303544; doi:10.3390/molecules28124589)
Supplement: Supplementary file 1 [file molecules-28-04589-s001.zip › molecules-2404337-supplementary.pdf]

Supplementary materials:

Nutritional and physicochemical properties of wild lingonberry (*Vaccinium vitis-idaea L.*) – effects of geographic origin

Dalia Urbonaviciene [1*], Ramune Bobinaite [1], Pranas Viskelis [1], Jonas Viskelis [1], Aistis Petruskevicius [1], Viktorija Puzeryte [1,2], Laima Cesoniene [2], Remigijus Daubaras[2], Linards Klavins [3], Ceslovas Bobinas [1]

A

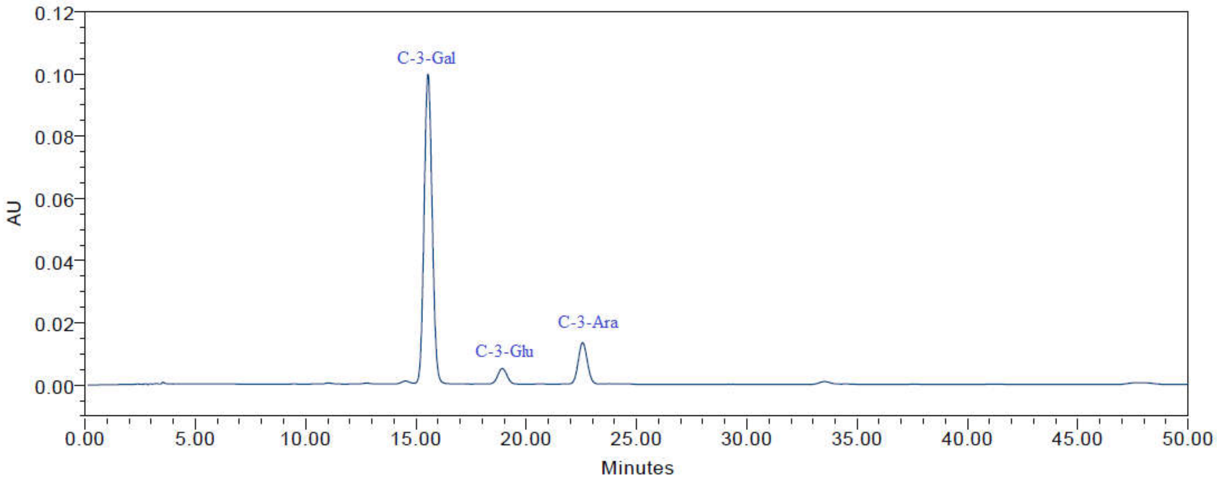

B

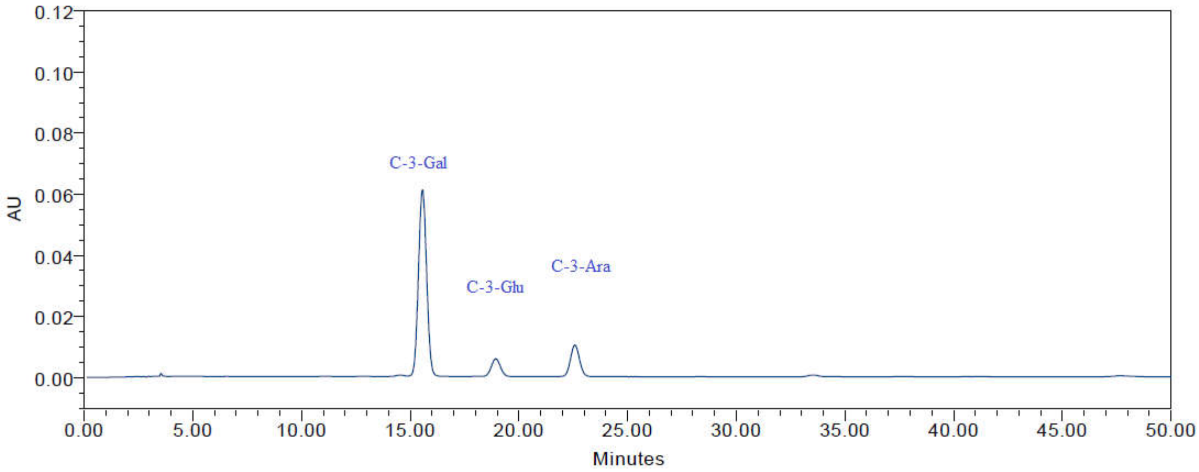

Figure S1. The anthocyanin profile determined by HPLC-DAD in wild *Vaccinium vitis-idaea* L. (A – NOR; B – LVA) Peaks: 1. Cyanidin-3-O-galactoside (C-3-Gal); 2. Cyanidin-3-O-glucoside (C-3-Glu); 3. Cya-nidin-3-O-arabinoside (C-3-Ara).

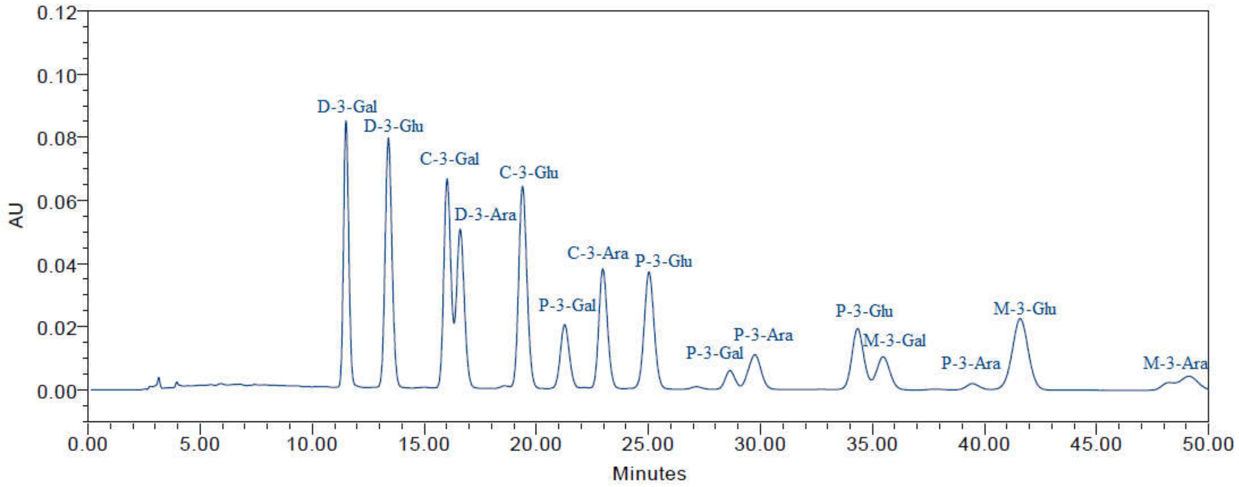

Figure S2. HPLC-DAD profiles of anthocyanins of European and US Pharmacopoeia Cromatography Reference Standard (CRS) Bilberry dry extract at 520 nm.
Peak indentification: 1) delphinidin-3-galactoside (D-3-Gal); 2) delphinidin-3-glucoside (D-3-Glu); 3) cyanidin-3-galactoside (C-3-Gal); 4) delphinidin-3-arabinoside (D-3-Ara); 5) cyanidin-3-glucoside (C-3-Glu); 6) petunidin-3-galactoside (P-3-Gal); 7) cyanidin-3-arabinoside (C-3-Ara); 8) petunidin-3-glucoside (P-3-Glu); 9) peonidin-3-galactoside (P-3-Gal); 10) pe-tunidin-3-arabinoside (P-3-Ara); 11) peonidin-3-glucoside (P-3-Glu); 12) malvidin-3-galactoside (M-3-Gal); 13) peonidin-3-arabinoside (P-3-Ara); 14) malvidin-3-glucoside (M-3-Glu); 15) mal-vidin-3-arabinodside (M-3-Ara)

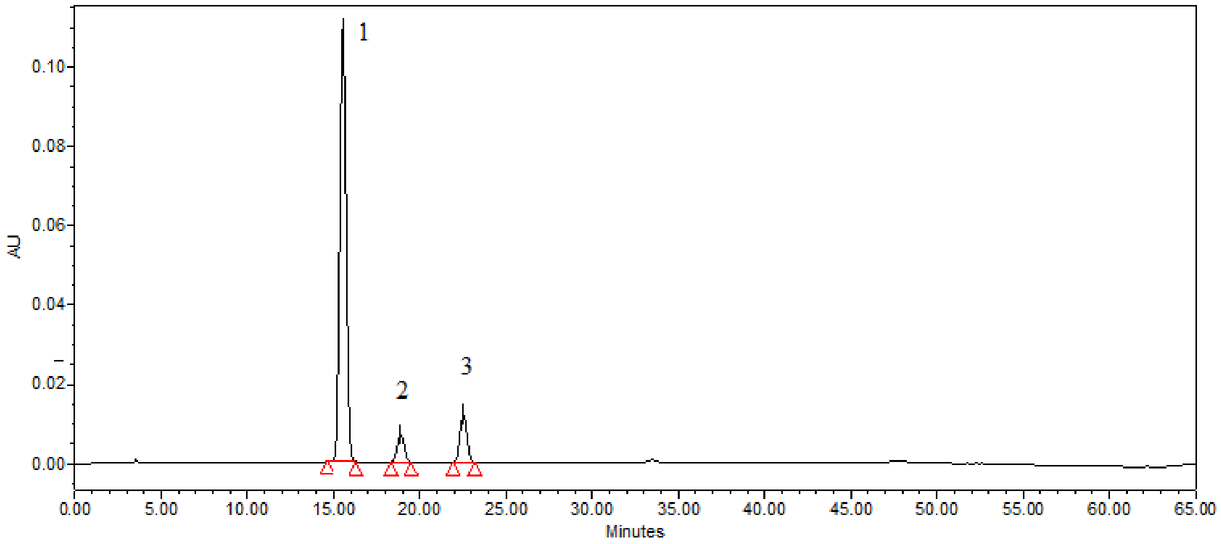

Figure S3. Integrated chromatogram

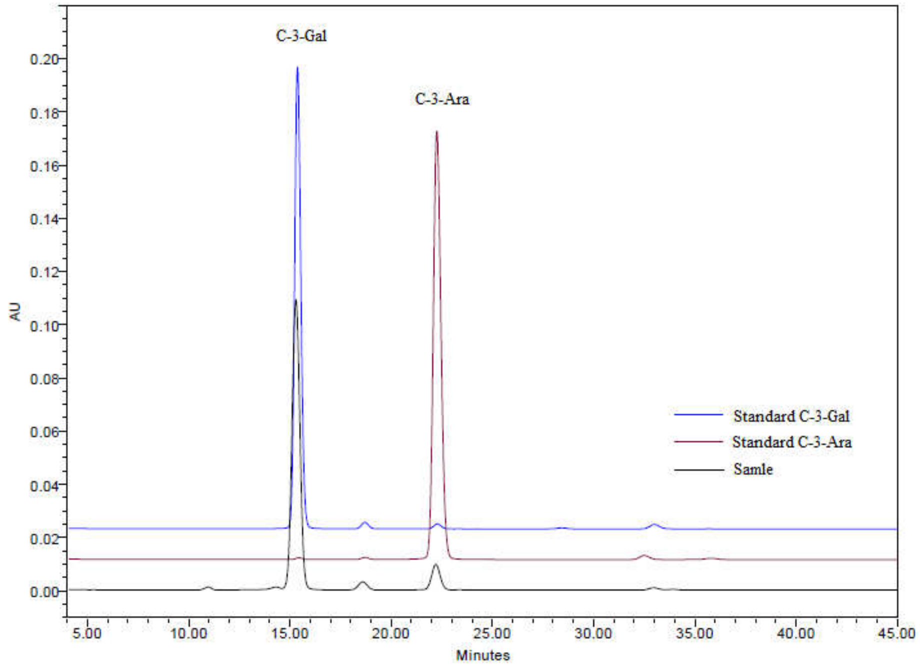

Figure S4. The external standards of cyaniding-3-O-galactoside (C-3-Gal), cyanidin-3-O-arabinoside (C-3-Ara) and sample of anthocyanin profile determined by HPLC-DAD in wild *Vaccinium vitis-idaea* L.

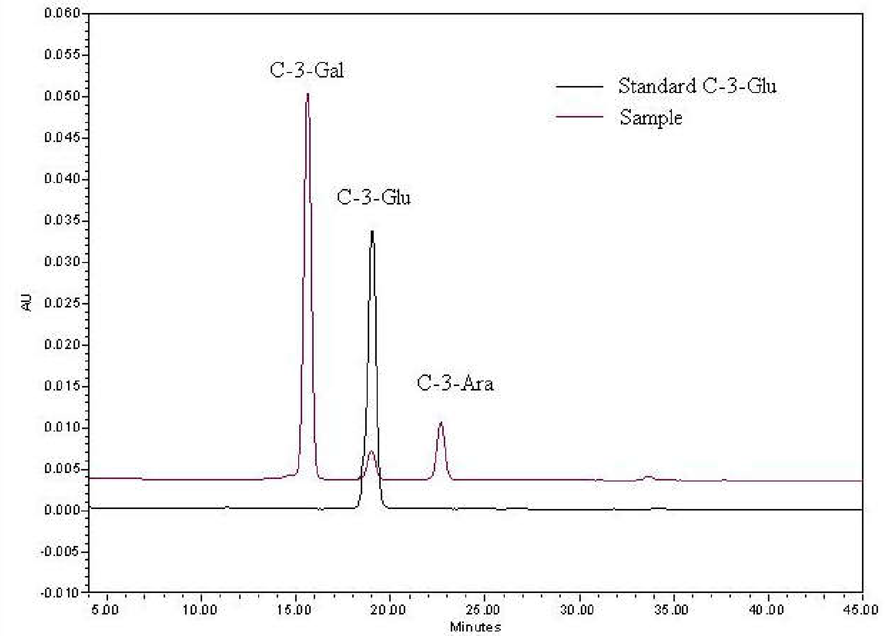

Figure S5. The external standard of cyanidin-3-O-glucoside (C-3-Glu) and sample of anthocyanin profile determined by HPLC-DAD in wild *Vaccinium vitis-idaea* L.
